# Case report: *Staphylococcus aureus* endocarditis in 2 premature newborns

**DOI:** 10.1097/MD.0000000000013549

**Published:** 2019-01-04

**Authors:** Marie Duperril, Stéphanie Rapin, Cécilia Vuillard, Isabelle Rayet, hugues Patural

**Affiliations:** a Neonatal Intensive Care Unit, Department of Pediatric Medicine, CHU de Saint-Etienne; b EA SNA-EPIS Research Laboratory, Jean Monnet University of Saint-Etienne, Saint-Etienne, France.

**Keywords:** infectious endocarditis, neonatal, *Staphylococcus aureus*, central venous cannula

## Abstract

**Rationale:**

Neonatal infectious endocarditis (IE) in a healthy heart is rare. The infectious agents most frequently found in newborns are *Staphylococcus aureus* and fungi. Infection at the site of central intravenous catheter is generally thought to be the cause of this pathology.

**Patient concerns:**

We present 2 cases of premature newborns whose condition is evolving positively. They presented *S aureus* endocarditis during their first week of life.

**Diagnosis:**

Modified Duke diagnostic criteria—from clinical, echocardiogram and microbiological findings—based on those used for adults, can be used for children and newborns, but the very low prevalence of neonatal IE often delays diagnosis. Diagnosis on the basis of transthoracic heart ultrasound requires an extension report, given the very high embolic risk. Intervention: In the large majority of cases, long-term antibiotic therapy efficaciously treats the infection, although sometimes surgery is necessary. These 2 newborns needed only antibiotic therapy.

**Outcome:**

Despite the various complications, especially embolic, these 2 children are followed and are doing well.

**Lessons:**

Long-term pediatric heart monitoring combined with prophylactic antibiotics are essential, according to the European Society of Cardiology guidelines.

## 1 Introduction

Infectious endocarditis (IE), rare in children and newborns, nevertheless represents a significant morbidity and mortality. It is defined as a bacterial infection of the valvular or nonvalvular endocardium. The characteristic lesion is the “vegetation”, fibrin and bacterial clusters, which destroy the heart structure and cause emboli.

The incidence of IE in children is lower than in adults, between 0.34 and 0.64 cases per 100,000 per year,^[1]^ but it appears to have been increasing in recent years.^[2]^ This is thought to be the result of increased life expectancy of children with congenital heart disease in developed countries (due to advances in neonatal care, with improved care for very premature babies) and increased use of central venous catheters in pediatric medicine.

Here we describe 2 cases of premature newborns, who presented with signs of infectious *Staphylococcus aureus* endocarditis during their first week of life. The umbilical cord catheter was suspected as the source of infection. Both cases had a positive clinical outcome after 6 weeks of intravenous antibiotic administration, without an increase in expected length of hospital stay.

## 2 Cases reports

Informed written consent was obtained from the patients’ parent for publication of the case details and accompanying image.

### 2.1 First case

Infant born prematurely by cesarean section, at 29 weeks + 6 d of gestational age, in the context of intrauterine growth restriction at the 10th percentile, and pre-eclampsia. At birth, adaptation to extrauterine life was suitable, with an APGAR score of 8^1’^/9^3’^/10^5’^/10^10’^. An intratracheal instillation of surfactant was done at H6 (Curosurf, 200 mg/kg) in front of a rise oxygen requirement, followed by noninvasive ventilation support. Parenteral feeding was started on the first day via umbilical venous catheter, followed by an epicutaneous central catheter at day 4. Enteral feeding was started together via nasogastric tube. No prophylactic antibiotics were given.

Laboratory assessment; at 24 hours of life; showed raised levels of C-reactive protein (CRP) at 36.8 mg/dL, initially suspected to be due to the surfactant therapy. On the third day, CRP levels had dropped to 14.1 mg/dL with a leukopenia (2.35 G/L) and a neutropenia (0.670 G/L). Microscopy, culture and sensitivity tests of gastric liquid and placenta were negative.

**Figure 1:**
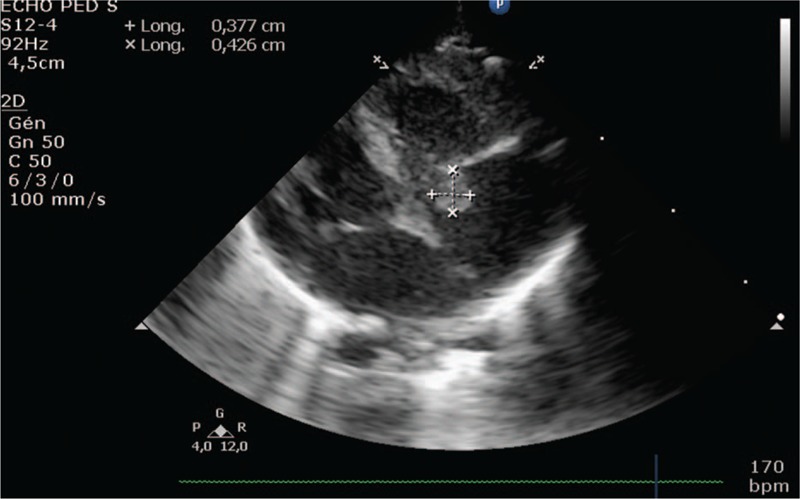
Vegetation on the tricuspid valve by echocardiography.

At day 5, the newborn presented a 37.9°C fever and iterative bradycardia that required an increased ventilation support. CRP remained below normal at 16.6 mg/dL after surfactant, and blood cultures showed the presence of *S aureus*. A triple antibiotic therapy, combining vancomycin (45 mg/kg/d), cefotaxime (100 mg/kg/d) and gentamicin (6.5 mg/kg/36 h) was started, then adapted according to sensitivity to oxacillin (200 mg/kg/d in 4 doses). Echocardiography showed oval vegetation 4.5 mm long on the tricuspid valve of and moderate valve leakage. Lumbar puncture was clear with few red cells (white blood cells = 56/mm3, red blood cells = 4700/mm3, CSF protein = 1.35 g/L, CSF glucose = 2.64 mmol/L). Cranial ultrasound and abdominal tomodensitometry were normal. Figure 1

Three days after the beginning of the infection, there was swelling of the neck and right arm at the site of the epicutaneous central catheter and Doppler showed a new thrombus of the superior vena cava (SVC). Heparin treatment was established (200 IU/kg/d) by peripheral intravenous access.

After 3 days of sensitivity-adapted antibiotics, the catheter was removed, with improving blood results. Antibiotics were continued on a peripheral intravenous catheter for 48 hours, followed by another contralateral epicutaneous central line. Blood cultures were sterile at day 11, following 4 days of oxacillin. This was continued for 6 weeks. The distal thrombus on the SVC disappeared after 8 days of heparin, like the cervical edema. Regular follow up with echocardiogram confirmed progressive reduction of the tricuspid vegetation, and its complete disappearance within 8 months. In the context of recurrent but temporary leukoneutropenia, a fetal-parental incompatibility assessment was done but found to be negative. The patient did well generally, with respiratory autonomy at day 34, complete enteral feeding at day 25, and brain imaging normal until corrected term.

### 2.2 Second case

Infant born prematurely by emergency caesarian section at 31 weeks + 1 d gestational age in the context of abnormal fetal heart rate.

The pregnancy was characterized by an acceleration of the middle cerebral velocity, linked with an antibody anti-D and anti-C alloimmunization with incompatible fetal genotyping, necessitating transfusion of fetal red blood cells. This led to an increase in the hemoglobin count from 7.3 g/dL to 13.5 g/dL.

At birth, adaptation to extrauterine life was difficult, with an APGAR score of 1^1’^/5^3’^/10^5’^/10^10’^, a lack of initial cry, and a bradycardia requiring physical stimulation and Néopuff ventilation with 21% then with 40% oxygen. The infant was intubated at 23 minutes of life before administration of intratracheal surfactant at 30 minutes of life. Anemia of 7.6 g/dL, an arterial pH of 7.32 and lactate levels of 3.2 mmol/L were found in the umbilical cord blood. The infant was transferred to the neonatal intensive care unit, where intensive light therapy and intravenous immunoglobulin was started in the context of severe anti-D alloimmunization. No prophylactic antibiotics were started.

Routine microbiology carried out at birth (external auditory canal, gastric liquid, and blood culture) were clear. CRP was at 3.3 mg/L on the third day of life. A parenteral feeding via an umbilical venous catheter was placed at birth then relayed by a jugular central venous catheter on the fifth day. Enteral feeding via nasogastric catheter was started on the first day of life.

On the fifth day of life, the newborn presented cardioplegic septic shock with a left ventricle dysfunction with anuria, requiring the use of intravenous fluids and vasopressors (noradrenaline and dobutamine).

Prophylactic antibiotics (amoxicillin, cefotaxime, and amikacin) were started for 24 hours, then linezolid (Zivoxid) and amoxicillin-clavulanic acid, following a blood culture showing the presence of *S aureus*. There was significant inflammation (CRP = 91 mg/dL, procalcitonin = 52.06 μg/L). The transthoracic echocardiogram showed a left intra-auricular vegetation, suggesting an IEs. On day 9 the initial antibiotics were adjusted with oxacillin at 200 mg/kg/d for 6 weeks. The last positive blood culture with *S aureus* was on the eighth day of life. Echocardiogram showed the disappearance of vegetation at day 41.

The child also received regular clinical and echographic neurological follow-up. At day 14, there were signs of bilateral occipital hyperechoic areas, suggesting a recent hemorrhagic or ischemic brain stroke. Head Magnetic Resonance Imaging (MRI) confirmed the presence of a right temporal hematoma (47×23×19 mm) and 2 occipital hematomas, (20×27×14 mm on the right side and 16×22×12 mm on the left), suggesting a brain abscess. We saw a partial reduction of the temporal and occipital hematomas and neurological checks remained normal during the neonatal period; auditory assessment showed no abnormality.

The newborn received mechanical ventilation support for 35 days, complicated by a left pneumothorax at day 7, which was drained for 7 days. This was followed by noninvasive ventilation for an additional 34 days. Complete respiratory autonomy, without oxygen, was obtained at 39 weeks + 3 days postconception.

Anemia and maternal-fetal alloimmunization were brought under control after 2 immunoglobin infusions, 3 transfusions of globular concentrate and one of platelet concentrate.

Multidisciplinary follow up was established at discharge from hospital, given the prematurity and the observed cerebral lesions.

## 3 Discussion

IE is rare in pediatric medicine during the neonatal period but represents significant morbidity and mortality, particularly in infants with congenital heart disease. Incidence of pediatric IE is about 0.6 cases per 100,000 per year (compared with 1.5 cases per 100,000 per year in adults.^[4,9]^

According to the literature, mortality associated with IE is in the range of 10% to 25%.^[1,6,7]^ Mortality is higher in premature infants.^[8]^ Factors affecting prognosis include the type of infectious agent,^[1,5,8]^ the location and site of the vegetation, concurrent pathologies, and complications linked to emboli.

Congenital heart disease represents the principal risk factor, linked to between 64% and 77% of IE cases.^[1,5]^ Complex cyanogenic heart disease presents the highest risk (8.2/1000 patients/y), followed by interventricular communications (2.4/1000 patients/y), tetralogy of Fallot (2.3/1000 patients/y) and aortic stenosis (2.0/1000 patients/y).^[6]^ Other major risk factors for IE are valvular prostheses, the presence of any synthetic material in the body, the presence of a central vascular catheter, and any surgical heart interventions.^[7]^

*S aureus* is the most commonly found infectious agent in pediatric IE in developed countries, in both congenital heart disease patients and in those with a healthy heart (40%–57% of cases ).^[8,10,11]^ In children with venous catheters, *S aureus* is the most common bacterium. Newborn cases are characterized by the predominance of *S aureus* and candida.^[12]^Other germs to note are *Enterococcus,* germs of the *Haemophilus parainfluenzae, Actinobacillus actinomycetemcomitans, Cardiobacterium hominis, Eikenella* species, and *Kingella kingae* (HACEK group).

In our 2 cases, infection was with methicillin-sensitive *S aureus,* occurring on the tricuspid valve in 1 case and the left intra-auricular in the other. Both patients were infants who had with an epicutaneous central catheter, the possible gate of infection. In the second case, the left intra-auricular vegetation could be explained by the presence of a patent foramen ovale. The IE was detected in the course of assessment to investigate neonatal infection. Hemodynamic stability could be quickly compromised, as in Case 2. It should be noted that a heart murmur was not found in either case, although this sign is one of the key modified diagnostic criteria of Duke. Cases of IE without a heart murmur have been noted in other studies (heart murmur was found in 21% of cases in Stockheim's study;^[13]^ in Del Pont and D Cicco's study^[14]^ they found a higher incidence of heart murmur (44%) in healthy hearts than in patients with heart disease (9%).

Splenomegaly is often found, as pleural effusion or pericarditis. Skin conditions such as Osler's nodes, Janeway lesions, digital clubbing or Roth's spots on the retina are not indicative but can be valuable in diagnosis. In newborns and children, transthoracic echocardiogram allows the detection of cardiac lesions in the majority of cases, with 93% sensitivity.^[15]^ Blood cultures are also a key diagnostic tool for IE, based on modified Duke criteria. In most cases, IE is present with complications: in 40% of children these are linked to emboli,^[3]^ with the migration of emboli to the lungs, brain, spleen, kidneys, the retina or, less commonly, peripheral vessels. Such emboli are often the cause of neurological complications (20%–40% of cases),^[4]^ such as ischemic or hemorrhagic cerebrovascular stroke, transient ischemic attacks, cerebral abscess, convulsions, and meningitis. Cardiac failure, sometimes severe, can cause complications, especially when found on the left side of the heart. According to the literature, 10% to 30% of children with IE present cardiac failure,^[16]^ which could be due to valve insufficiency, myocardic abscess, significant obstructive vegetation, or coronary emboli. Other possible complications are blood-related (disseminated intravascular coagulation), vascular (mycotic aneurisms), renal (renal failure), splenic (abscess), myocarditis, or pericarditis, pleural (effusions) and peritoneal. In our 2 examples, one of the patients presented with a complication consisting of thrombus within the SVC, requiring anticoagulation using heparin. The other patient presented with cerebral hemorrhage detected fortuitously during a transfontanelar ultrasound checking and confirmed by head MRI.

The duration of intravenous antibiotic treatment of IE for children on a healthy heart is on average 6 weeks. For endocarditis without complications, due to *S viridans*, sensitive to penicillin, antibiotic treatment for 2 to 4 weeks can be appropriate.^[2]^ In the case of endocarditis on the left heart, with *S aureus,* sensitive to oxacillin, patients should receive 4 weeks of intravenous antibiotics.^[17]^ In our two observations, the newborns received 6 weeks intravenous antibiotics—oxacillin. One of the patients received a double antibiotherapy by oxacillin and rifampicin and the other one received 5 doses of gentamicin. However, in the large majority of cases of IE with *S aureus*, sensitive to methicillin, the use of an aminoglycoside is not indicated, as it has shown no improvement in prognosis and an increased risk of nephrotoxicity and ototoxicity.^[18]^

Prophylaxis against IE is required for patients who have a replacement heart valve or other prosthetic apparatus, patients with a history of IE and those with congenital heart disease. Invasive dental treatment in these cases requires administration of amoxicillin 50 mg/kg (maximum 2 g), 30 to 60 min before an intervention.

In neonatal intensive care, an active surveillance culture and decolonization of Staphylococcus aureus could decrease the rate of infections.^[19]^

## 4 Conclusion

IE is rare in newborns and children. Rapid echocardiographic diagnosis and advances in treatment have led to a significant reduction in mortality in developed countries, but mortality remains high. To diagnose IE, a high index of suspicion is necessary, especially where a central venous cannula or other risk factor is present, and extensive investigation is required to detect any associated complications.

## Author contributions

**Supervision:** Rapin Stéphanie, Vuillard Cécilia, Rayet Isabelle, Patural Hugues.

**Validation:** Patural Hugues.

**Writing – original draft:** Duperril Marie.
